# Low Incidence Rate of Opportunistic and Viral Infections During Imatinib Treatment in Chronic Myeloid Leukemia Patients in Early and Late Chronic Phase.

**DOI:** 10.4084/MJHID.2011.021

**Published:** 2011-05-16

**Authors:** Massimo Breccia, Corrado Girmenia, Roberto Latagliata, Giuseppina Loglisci, Michelina Santopietro, Vincenzo Federico, Luigi Petrucci, Alessandra Serrao, Adriano Salaroli, Giuliana Alimena.

**Affiliations:** Department of Cellular Biotechnologies and Hematology, Sapienza University, Rome, Italy

## Abstract

**Background:**

Imatinib has become first line therapy in chronic myeloid leukemia patients. Little is known about the infective consequences during the treatment with this drug in large series of chronic phase patients.

**Material and methods:**

From January 2001 to September 2006 we treated with imatinib 250 patients in first line (early CP) or after interferon failure (late CP), out of clinical trials and recorded all the bacterial and viral infections occurred.

**Results:**

We recorded a similar incidence of bacterial and viral infections both in first line and late CP patients (respectively, 16% and 13%) during 3.5 years of follow-up. Analysis of presenting features predisposing to infections revealed differences only in late CP patients, with elevated percentage of high Sokal risk patients and a more longer median time from diagnosis to start of imatinib.

**Conclusions:**

Opportunistic infections and reactivation of Herpes Zoster are observed during imatinib therapy at very low incidence.

## Introduction:

Targeted inhibition of BCR-ABL with imatinib mesylate has become the standard therapy for patients with chronic myeloid leukaemia (CML) where it induces a complete cytogenetic response in more than 80% of newly diagnosed patients.^1^ A recent 8-year follow-up of IRIS study showed an overall survival (OS) of 85% and event-free survival (EFS) of 81%.^2^ Several years after the introduction of imatinib in clinical practice no significant major incidence of infections was reported. However, different evidences that imatinib can impair several cellular functions involved in the immune response have been observed, in particular in cell-mediated immunity.^3^–^5^ We refer here on the incidence of infectious episodes observed during imatinib treatment in our large series of early and late chronic phase CML patients.

## Patients and Methods:

All consecutive patients with Ph+ CML in chronic phase out of clinical trials who started with imatinib therapy during the period from January 2001 to September 2006 were included in the study. The observation was interrupted on December 2009 and patients lost to follow-up were considered until last visit. Overall 250 patients were considered: 150 patients had received prior therapy with Interferon (IFN) for a median of 24 months (range 5–68) and were considered in late chronic phase (CP) and, whereas 100 patients received imatinib soon after diagnosis (early CP). No differences were observed regarding Sokal risk: in early CP patients at baseline, 45% were low risk, 24% intermediate and 3% were high risk. In late CP group, Sokal risk at the time of imatinib start revealed 43% of patients as low risk, 50% intermediate and 6.6% as high risk. Before starting imatinib, all patients had baseline evaluation including physical examination, complete blood cell count, renal and hepatic function tests, serum protein electrophoresis, serum levels of IgG, IgA and IgM, bone marrow (BM) aspirate with cytogenetic and molecular analyses, chest X ray and cardiac evaluation. Physical examination and complete blood cell count were performed weekly for the first month, then monthly. During treatment, all side effects possibly related to drug and all infective episodes, were evaluated and collected by medical staff. Only clinically and microbiologically documented infections were included. Febrile episodes of unknown origin in non-neutropenic patients (ANC > 500/cmm), upper respiratory tract syndromes possibly related to a viral infection and localized Herpes simplex were not considered in the analysis.

## Results:

Overall, 36 clinically or microbiologically documented infections that required antibacterial or antiviral therapy and discontinuation of imatinib were recorded in 35 patients (incidence 14%). The characteristics of the infective episodes are summarized in table 1.

In the series of 100 CP patients who received imatinib as first line therapy, with a median follow-up of 3.5 years, we recorded 17 infectious episodes in 16 patients (incidence 16%). They accounted for 0.02 infectious episodes per 1000 patient days. All the infective episodes occurred at a median time of 13 weeks (range 9–26) from the onset of imatinib treatment. No episode was associated to deep neutropenia (absolute neutrophyl count < 500/cmm). Median gammaglobulin dosage was 0.82 g/dl (range 0.7–2), with only 1 patient presented with a slightly inferior dosage. Herpes zoster and pneumonia represented the two more frequently observed infections occurring in 7% and 4% of patients, respectively. In all patients who developed Herpes zoster a reduction of lymphocyte count (median lymphocytes count 0.6 x 10^9^/l, range 0.2–1.1, compared to 0.9 x 10^9^/l of patients who did not developed viral infections) was evidenced at the time of viral infection (median time of development 11 weeks), in the absence of induced leukopenia and serum Ig level (0.9 g/dl). One out of the 4 subjects who developed pneumonia presented two additional episodes of fever with radiological evidence of pulmonary infiltrate diagnosed as tuberculosis. This second infection occurred soon after underlying hematologic disease progression to accelerated phase.

In the series of 150 patients treated with imatinib after resistance/intolerance to interferon, observed for a median follow-up of 4 years, we recorded 19 infective episodes (13%). They accounted for 0.003 infectious episodes per 1000 patient days. Three episodes of pneumonia and 2 urinary tract infections occurred during neutropenia phase. Median gammaglobulin dosage was 0.93 g/dl (range 0.6–2.1), with only 2 patients presented with a slightly inferior dosage. Fourteen late CP patients developed a Herpes zoster infection during treatment with imatinib (median time of development 14 weeks): these patients, as observed in the cohort of early CP patients, had a significant reduction of lymphocyte count at the time of viral infection (median lymphocytes count 0.8 x 10^9^/l, range 0.2–1.2, compared to 1.1 x 10^9^/l of patients who did not developed viral infections), in the absence of leukopenia and serum Ig level (0.8 g/dl). The infective episodes developed at a median time of 20 weeks (range 10–28) from the onset of imatinib treatment and all occurred during neutropenia induced by the drug, differently from what observed in early CP patients. No infection-related death occurred and all infections resolved in both groups of patients. Presenting features of patients who developed infections and of those who did not were compared (Table 2). In the group of subjects in early CP there were no statistically significant differences between patients who developed infections and patients who did not. In patients in late CP a longer interval of time between diagnosis and the start of imatinib (17 vs 9 months, p=0.02) and a prevalence of high Sokal risk (p=0.03) was observed in patients who developed an infectious complication.

## Discussion:

Clinical and laboratory findings seem to show that imatinib can impair several cellular functions involved in the immune response. Two different studies referred on a reduction of the immunoglobulin levels during imatinib treatment for CML and for gastrointestinal stromal tumors (GIST).^6^,^7^ In the report of Steegmann et al^6^ only a slight decrease of Ig levels in imatinib treated patients who had became resistant or intolerant to interferon-alpha was documented: the authors cannot rule out the possibility of an influence by the prior therapy and suggested an impairment of B-lymphocytes or a mediated effect through the inhibition of ABL kinase. Santachiara et al^7^ reported on 87 late CP patients treated with imatinib who presented a reduction of Ig levels; these patients were treated with imatinib after IFN for CML or were affected by GIST. The authors did exclude that this effect had to be ascribed to interferon therapy and suggested that it had to be considered the consequence of the imatinib treatment. CML patients in CP are at low infectious risk compared to patients in more advanced disease phases, however the epidemiological impact of infectious complications in the imatinib era is unknown as infections have not been enclosed in the safety analysis of large imatinib studies.^2^,^8^ Few reports on non-viral infections have been published during imatinib therapy and anecdotic cases of pulmonary nocardiosis, pulmonary tuberculosis, fungal pneumonia and listeria meningitis have been reported.^9^–^12^ In two of these observations, the infections were concomitant to lymphopenia and monocytopenia. On the contrary, viral infections, particularly those caused by herpes viruses, seem to have a higher epidemiological impact.^13^–^15^ Mattiuzzi et al^13^ reported on a frequency of 2% of VZV reactivation in CML patients treated with imatinib: baseline features of these subjects did not differ significantly from those of patients who did not develop VZV infection, except for the time from diagnosis to imatinib treatment and the number of prior therapies. Few case reports reported on the occurrence of other viral infections during imatinib therapy.^14^–^16^ In particular, the case of a fatal HHV8 infection in a CML patient in complete molecular remission after imatinib was recently reported.^15^ In our experience, bacterial infection had a very low epidemiological and clinical impact but VZV disease occurred more frequently than previously reported.^13^ Contrary to the study by Mattiuzzi et al,^13^ all patients enclosed in our series were out of clinical trials, probably with less favourable clinical characteristics. In fact, all patients who developed the viral infection were aged over 50 years and had comorbidities, such as diabetes and/or cardiac disease, conditions which contraindicated the inclusion in clinical trials. We did not find differences in the VZV incidence between early and late CP patients (7% vs 9%). In any case, VZV infections accounted for only 0.04 infectious episodes per 1000 patient days in the overall population, therefore prophylaxis of VZV infection may not be recommended in this setting, considering also the limited extent of the disease and the prompt response to therapy. As to bacterial infections in late CP group, we found that a longer interval of time between diagnosis and the start of imatinib and a prevalence of high Sokal risk were prognostic adverse events compared to early CP group: a possible explanation is that previous treatment and high Sokal risk may influence the rate of infections due to the possible scarce reserve of Ph negative cells. Infectious episodes appeared concentrated in the first period of treatment: this is in line with data from trials,^2^ in which toxicity was observed in the first year of treatment and new events did not occur lately, again probably related to the initial massive reduction of leukemic burden.

## Conclusions:

We observed that opportunistic infections are an unusual complication also in a “real life” population of CP - CML patients under imatinib therapy. No life threatening and easy to treat Herpes zoster reactivations represents the most frequently observed infectious complications.

## Figures and Tables

**Table 1.** Characteristics of patients and of infective episodes.

**Characteristics**			

	**Early chronic phase**	**Late chronic phase**	**Total population**

**Number of patients**	100	150	250

**Age years, median (range)**	54 (22–79)	56 (21–82)	55 (21–82)

**Sex M/F, number of patients**	66/34	82/68	148/102

**Follow up years, median (range)**	3.5 (2–7)	4 (2.2–9)	4.2 (2–9)

**Time from start of imatinib therapy and first infectious episode, weeks (range)**	13 (9–26)	20(10–28)	15(9–28)

**Neutropenia (ANC< 500/cmm) during imatinib therapy, number of patients (%)**	14 (14%)	12 (8%)	26 (10%)

**Infections, number of patients (%)**			
**Febrile neutropenia of unknown origin**	4	5	9
**Pneumonia**	4*	3	7
**Pericarditis**	1	/	1
**Urinary tract infection**	3	2	5
**Orchitis**	1	/	1
**Herpes zoster**	7	14	21
**Total patients with infection**	16 (16)	19 (13)	35 (14)

* One patient developed two episodes of pneumonia. In the second episode pulmonary tuberculosis was diagnosed.

**Table 2.** Comparison of patients who developed infections and patients who did not

**Feature**	**Patients in early chronic phase**			**Patients in late chronic phase**		

	**Patients with infections**	**Patients without infections**	**p**	**Patients with infections**	**Patients without infections**	**p**

**Median age**	54	56	0.756	55	57	0.654

**Sex**			0.456			0.876
**Male**	7	26		10	70	
**Female**	9	30		9	61	

**Sokal risk**			0.355			0.03
**Low**	10	35		4	60	
**Intermediate**	4	20		7	69	
**High**	2	1		8	2	

**Median time from diagnosis to start of imatinib (months)**	1.5	2	0.233	17	9	0.02
